# Diversion ostomy improves treatment tolerance, conversion surgery, and survival compared with self-expanding metal stenting in initially unresectable obstructive colorectal cancer

**DOI:** 10.3389/fonc.2026.1881728

**Published:** 2026-07-15

**Authors:** Fengbin Cai, Weiming Jiang, Huali Cai, Bo Hu

**Affiliations:** 1 Department of Gastrointestinal Surgery, Xiamen Humanity Hospital, Fuiian Medical University, Xiamen, China; 2 Department of Thyroid and Breast Surgery, Xiamen Humanity Hospital, Fuiian Medical University, Xiamen, China

**Keywords:** conversion therapy, diversion ostomy, obstructive colorectal cancer, self-expanding metal stent, subsequent resection

## Abstract

**Background:**

The optimal decompression strategy for patients with initially non-resectable obstructive colorectal cancer undergoing conversion intent treatment remains unclear. This study compared diversion ostomy (DO) and self-expanding metal stent (SEMS) placement.

**Methods:**

Data from 115 patients with initially non-resectable obstructive colorectal cancer, who were treated between June 2021 and June 2025, were retrospectively reviewed. Sixty-two patients underwent DO, and 53 underwent SEMS as the initial decompression strategy. After decompression, patients received oxaliplatin and oral capecitabine (i.e., “CAPEOX”)- or folinic acid, fluorouracil, and oxaliplatin (i.e., “FOLFOX”)-based systemic therapy with targeted therapy, with or without immunotherapy, according to molecular and immunohistochemical findings. Clinical outcomes, nutritional and inflammatory indices, tumor response, subsequent resection, and overall survival (OS) were compared between the two groups.

**Results:**

DO was associated with a significantly higher rate of achievement of Colorectal Obstruction Scoring System (CROSS) score 3 than SEMS (85.5% versus [vs.] 47.2%; P<0.001), and a higher number of chemotherapy cycles (median, 6 vs. 2; P<0.001). Post-treatment nutritional and inflammatory profiles were more favorable in the DO group, with higher Prognostic Nutritional Index (≥45) rates and lower platelet-to-lymphocyte and neutrophil-to lymphocyte ratios (all P ≤ 0.005). The objective response and subsequent resection rates were significantly higher in the DO group than that in the SEMS group (69.4% vs. 30.2%; P<0.001), as was the subsequent resection rate (69.4% vs. 30.2%; P<0.001), respectively. Kaplan–Meier analysis revealed significantly longer OS in the DO group, with a median survival of 27.8 vs. 10.3 months (P<0.0001). In multivariate analysis, SEMS remained independently associated with worse OS than DO (hazard ratio 2.231 [95% confidence interval 1.345–3.842; P = 0.001).

**Conclusions:**

In patients with initially non-resectable obstructive colorectal cancer treated with conversion intent, DO was associated with better decompression, improved treatment tolerance, higher resection rates, and longer survival than SEMS placement.

## 1 Introduction

Colorectal cancer is among the most common malignant malignancies worldwide and a leading cause of cancer-related mortality (1). Although radical resection is the standard treatment for localized disease, a substantial proportion of patients present with locally advanced or metastatic tumors at the initial diagnosis, making curative surgery non-feasible (2, 3). Among these patients, some are still considered to be potentially convertible after effective systemic therapy and may eventually become candidates for radical resection (4, 5).

Malignant colonic obstruction is a common and serious complication of advanced colon cancer, often requiring urgent intervention (6–8). Treatment is particularly challenging in patients with potentially resectable obstructive colon cancer. The initial intervention must not only relieve obstruction but also preserve the opportunity for systemic therapy and subsequent conversion surgery. For patients without obstruction, current guidelines recommend upfront systemic therapy with repeated reassessment of response and resectability in a multidisciplinary team (MDT) setting (6, 9–14).However, in patients with an obstruction(s), systemic therapy is often not feasible until bowel decompression is achieved.

Several strategies are available to relieve malignant colonic obstruction. Although palliative colectomy has traditionally been performed, it may be associated with more sever surgical trauma, delayed recovery, and interruption of subsequent systemic treatment (15–17). As such, palliative decompression has been increasingly adopted as a bridging strategy before conversion therapy. The two main decompression options are diversion ostomy (DO) and self-expanding metal stent (SEMS) placement (18, 19).

SEMS offers the advantages of rapid decompression and lower initial invasiveness, and is recommended for selected patients with malignant large bowel obstruction (20, 21). However, its long-term value remains controversial, particularly in patients expected to undergo prolonged systemic therapy because complications, such as perforation, migration, re-obstruction, and inadequate expansion, may compromise subsequent treatment (22). In contrast, DO is initially more invasive, but may yield more durable decompression and better support for subsequent treatment. However, the optimal priorities for these two strategies remain unclear.

Most previous studies have focused mainly on short-term outcomes, and evidence directly comparing DO and SEMS in initially unresectable obstructive colorectal cancer treated with conversion intent remains limited (18, 19, 23). As such, the present study aimed to compare the clinical outcomes of DO and SEMS placement in this setting, with a particular focus on decompression efficacy, treatment tolerance, tumor response, subsequent resection, and survival outcomes.

## 2 Methods

### 2.1 Patient selection

The medical records of patients with initially non-resectable obstructive colorectal cancer, who were treated at Xiamen Humanity Hospital (Fujian, China) between June 2021 and June 2025, were retrospectively reviewed. Eligible patients were identified from the institutional electronic medical record system. The inclusion criteria were as follows: histologically confirmed colorectal adenocarcinoma; radiologically and/or endoscopically confirmed primary tumor-related colonic obstruction at initial presentation; initially non-resectable disease as determined by an MDT; and treatment with either DO or SEMS placement as the initial decompression strategy. Eligible patients were included if they had colorectal adenocarcinoma confirmed histologically either before decompression or during the subsequent diagnostic or surgical course. In patients with severe obstruction in whom pre-intervention colonoscopy and biopsy were not feasible, the initial diagnosis was based on clinical manifestations, radiological evidence of primary colorectal tumor-related obstruction, and MDT assessment, with histological confirmation obtained subsequently from biopsy or surgical specimens. Malignant bowel obstruction was defined as clinically evident obstructive symptoms, including abdominal distension, abdominal pain, vomiting, constipation, or inability to tolerate oral intake, caused by a primary colorectal tumor and confirmed by abdominal computed tomography and/or endoscopic evaluation. Initially unresectable colorectal cancer was defined as disease that was not considered suitable for curative resection at initial presentation because of locally advanced invasion, peritoneal metastasis, hepatic metastasis, distant lymph node metastasis, or other non-curative factors, as determined by the institutional multidisciplinary team. Patients with non-malignant obstruction, recurrent colorectal cancer, previous anti-tumor treatment before admission, incomplete clinicopathological data, or other decompressive procedures were excluded. Patients with primary rectal cancer requiring a rectal cancer-specific neoadjuvant chemoradiotherapy or total neoadjuvant therapy strategy were excluded to reduce treatment heterogeneity. The present cohort therefore focused on obstructive colon or rectosigmoid colorectal cancers managed with systemic conversion-intent therapy after initial decompression. This study was approved by the Ethics Committee of Xiamen Humanity Hospital. All procedures were performed in accordance with the principles of the Declaration of Helsinki.

### 2.2 Treatment strategies

Treatment decisions were made by the institutional MDT comprising colorectal surgeons, medical oncologists, radiologists, endoscopists, and other relevant specialists. Treatment selection was not randomized. In our institutional practice, obstruction severity was an important consideration when selecting the decompression strategy. Patients with more severe obstruction were preferentially treated with diversion ostomy, whereas patients with relatively less severe obstruction were considered for SEMS placement. Both right-sided and left-sided tumors were included. SEMS placement was considered only in selected patients without suspected perforation or peritonitis and when the obstructive lesion was judged technically accessible by experienced endoscopists. For right-sided colonic obstruction, SEMS placement was performed only when safe guidewire passage and stent deployment were considered feasible under endoscopic and fluoroscopic guidance. Patients with complete obstruction, severe bowel dilation, unstable general condition, suspected perforation, or lesions unsuitable for safe stent placement were preferentially treated with diversion ostomy. The severity of obstruction was assessed in accordance with the Colorectal Obstruction Scoring System (CROSS) (24).

In the present cohort, patients initially underwent ileostomy in the DO group or endoscopic colonic SEMS placement in the SEMS group. In the DO group, diversion was performed as ileostomy or colostomy according to tumor location, bowel dilation, intraoperative findings, and the surgeon’s judgment. Ileostomy was frequently selected because it allowed rapid decompression while avoiding extensive manipulation of a dilated or tumor-involved colon, and it also facilitated later radical colectomy and simultaneous stoma closure when conversion surgery became feasible. Experienced endoscopists performed all SEMS procedures. Immediately after stent placement, stent expansion was assessed to ensure adequate deployment and satisfactory luminal patency, and the patients received conversion therapy consisting mainly of oxaliplatin and oral capecitabine (CAPEOX)- or folinic acid, fluorouracil, and oxaliplatin (FOLFOX)-based chemotherapy (25). The use of targeted therapies, including bevacizumab or cetuximab, as well as immunotherapy, was determined based on genomic testing and immunohistochemical findings. The response to conversion therapy was evaluated every two cycles.

Because there is currently no universally accepted definition for resectability after conversion therapy, resectability was determined based on the clinical judgment of the treating physicians and MDT assessment at the time of optimal tumor response (complete response or partial response). Once the MDT considered the tumor to be resectable, the patient underwent radical right or left hemicolectomy. After adequate postoperative recovery, systemic therapy was resumed as planned. In the DO group, stoma closure was performed simultaneously with radical colectomy.

The number of pre- and postoperative chemotherapy cycles was adjusted according to treatment tolerance and disease status. In general, preoperative chemotherapy is administered for four to six cycles, whereas postoperative chemotherapy is continued for six to eight cycles until unacceptable toxicity, intolerable adverse events, or MDT-confirmed resectability is achieved. For metastatic lesions, all patients were evaluated by the MDT for resectability and treated with synchronous or staged surgical resection, radiofrequency ablation, or other local treatment modalities when appropriate.

### 2.3 Data collection

Clinicopathological data were collected from the institutional electronic medical record system. The baseline variables included age, sex, Performance Status (PS), body mass index (BMI), obstruction severity, nutritional and inflammatory indices, clinical tumor stage, and incurable factors. Obstruction severity was assessed according to the CROSS. Nutritional status was evaluated using the Prognostic Nutritional Index (PNI) and BMI, whereas systemic inflammatory status was assessed using the platelet-to-lymphocyte ratio (PLR) and neutrophil-to-lymphocyte ratio (NLR) (26, 27). According to cutoff values commonly used in previous studies evaluating nutritional and systemic inflammatory markers in gastrointestinal malignancies and colorectal cancer, patients were categorized as follows: PNI <45 and ≥45, PLR <162 and ≥162, and NLR <2.5 and ≥2.5. These thresholds were selected based on previous literature (26, 27). Post-treatment clinical outcomes were evaluated at three months after the initial decompression procedure. At this time point, CROSS score, BMI, PNI, PLR, and NLR were reassessed. CROSS score 3 achievement was defined strictly as a point-in-time assessment at three months after decompression, indicating the ability to tolerate a low-residue or full diet without obstructive symptoms. It was not defined by chemotherapy continuation or the number of chemotherapy cycles. Radiological tumor response was also evaluated at the three-month post-decompression assessment according to RECIST criteria after systemic therapy. For patients who underwent subsequent resection before or around this time point, the latest available preoperative evaluation was used.

The clinical outcomes after treatment included the achievement of CROSS 3, number of chemotherapy cycles, post-treatment BMI, post-treatment PNI, PLR, NLR, radiological tumor response, and rate of subsequent resection. Tumor response was evaluated according to the Response Evaluation Criteria for Solid Tumors (i.e., “RECIST”) criteria (28). The objective response rate (ORR) was defined as the proportion of patients who achieved complete response (CR) or partial response (PR). Technical success of SEMS placement was defined as successful deployment of the stent across the obstructive lesion with adequate radiological and/or endoscopic expansion. Early clinical success was defined as relief of obstructive symptoms within 72 hours after SEMS placement without the need for emergency surgery. Technical success of SEMS placement was defined as successful deployment of the stent across the obstructive lesion with adequate radiological and/or endoscopic expansion. Early clinical success was defined as relief of obstructive symptoms within 72 hours after SEMS placement without the need for emergency surgery. In contrast, CROSS score 3 achievement was evaluated at three months after decompression and was defined solely according to oral intake and obstructive symptoms at that time point. Specifically, CROSS score 3 indicated the ability to tolerate a low-residue or full diet without obstructive symptoms. Chemotherapy continuation and the number of chemotherapy cycles were analyzed separately as treatment-process outcomes and were not components of CROSS score 3 achievement.

For patients who subsequently underwent surgery, operative and pathological outcomes were recorded, including resection margin status, pathological response, pathological T stage, and pathological N stage.

### 2.4 Follow-up

Patients were followed-up through outpatient visits, inpatient records, and telephone interviews. The follow-up continued until death or the last follow-up in March 2026. Overall survival (OS) was defined as the interval from the date of initial treatment to death from any cause or the last follow-up.

### 2.5 Statistical analysis

All statistical analyses were performed using SPSS version 22.0 (IBM Corp., Armonk, NY, USA) for Windows (Microsoft Corp., Redmond, WA, USA). Continuous variables are expressed as median (range) and were compared using the Wilcoxon rank-sum test or Mann–Whitney U test, as appropriate. Categorical variables are expressed as number (percentage) and were compared using Pearson’s chi-squared test or Fisher’s exact test, as appropriate.

Survival curves were estimated using the Kaplan–Meier method and compared using the log-rank test. Variables associated with overall survival were first analysed using univariate Cox proportional hazards regression and those with potential prognostic significance were subsequently entered into a multivariate Cox regression model to identify independent prognostic factors. Hazard ratio (HR) and corresponding 95% confidence interval (CI) were calculated. All statistical tests were two-sided, and differences with P < 0.05 were considered to be statistically significant.

## 3 Results

### 3.1 Baseline characteristics

Data from 115 patients with initially non-resectable obstructive colorectal cancer were included in this study: 62 underwent DO and 53 underwent SEMS placement. There were no significant differences between the two groups in terms of age, sex, PS, BMI, PNI, PLR, NLR, clinical T stage, or non-curative factors (all P>0.05) (**Table 1**). The median age was 58 years (range, 31–73 years) in the DO group and 67 years (range, 31–83 years) in the SEMS group (P = 0.073). The proportion of males was 62.9% and 62.3%, respectively (P = 0.944). The distribution of PS scores 0, 1, and 2 was n = 5, n = 27, and n = 30, respectively, in the DO group, and n = 3, n = 10, and n = 30, respectively, in the SEMS group (P = 0.081). In addition, the proportions of patients with PNI < 45 were 69.4% and 69.8% (P = 0.958), those with PLR ≥ 162 were 61.3% and 62.3% (P = 0.915), and those with NLR ≥ 2.5 were 74.2% and 75.5% (P = 0.875) in the DO and SEMS groups, respectively (**Table 1**).

**Table 1:** Baseline patients characteristics.

Variable	DO (n=62)	SEMS (n=53)	*P* *value*
Age (year)	58 (31-73)	67 (31-83)	0.073
Sex (male/female)	39/23(62.9/37.1)	33/20(62.3/37.7)	0.944
PS (0/1/2)	5/27/30(8.0/43.5/48.4)	3/20/30(5.7/37.7/56.6)	0.656
BMI	21.6 (16.2-25.4)	22.2 (16.2-26.2)	0.656
CROSS (0/1/2)	62/0/0(100/0/0)	0/53/0(0/100/0)	<0.001
PNI			0.958
<45	43(69.4)	37(69.8)	
≥45	19 (30.6)	16(30.2)	
PLR			0.915
<162	24(38.7)	20(37.7)	
≥162	38(61.3)	33(62.3)	
NLR			0.875
<2.5	16 (25.8)	13(24.5)	
≥2.5	46(74.2)	40(75.5)	
cT			0.556
T2	2(3.2)	1(1.9)	
T3	10(16.1)	12(22.6)	
T4a	47(75.8)	35(66.0)	
T4b	3(4.8)	5(9.4)	
cN (+)	62 (100)	53 (100)	–
Non-curable factor			
Infiltration to adjacent organs	3(4.8)	5(9.4)	0.334
Peritoneal metastasis	28(45.2)	27(50.9)	0.536
Hepatic metastasis	8(12.9)	6(11.3)	0.796
Distant lymph node metastasis	36(58.1)	26(49.1)	0.334

PS, performance status; DO, diversion ostomy; SEMS, self-expanding metal stent; CROSS, Colorectal Obstruction Scoring System; PNI, Prognostic Nutritional Index; BMI, Body mass index; PLR, Platelet to lymphocyte ratio; NLR, Neutrophil to lymphocyte ratio.

Notably, the distribution of CROSS scores differed significantly between the two groups: all patients in the DO group had a CROSS score of 0, whereas all those in the SEMS group had a CROSS score of 1 (P < 0.001) (**Table 1**).

### 3.2 Clinical outcomes after treatment

At the three-month post-decompression assessment, CROSS score 3 was significantly more frequent in the DO group than in the SEMS group (53/62 [85.5%] vs. 25/53 [47.2%]; P < 0.001). In the SEMS group, technical success was achieved in 51/53 patients (96.2%), and early clinical success was achieved in 45/53 patients (84.9%). During subsequent treatment, re-obstruction or stent occlusion occurred in 14 patients (26.4%), stent migration in 4 patients (7.5%), and perforation in 2 patients (3.8%). Reintervention was required in 16 patients (30.2%), including repeat endoscopic intervention, decompressive surgery, or emergency management according to the clinical condition. These events partly explained why the three-month CROSS score 3 achievement rate in the SEMS group was lower than the early clinical success rate. Chemotherapy exposure was analyzed separately from CROSS score assessment. Patients in the DO group also underwent significantly more chemotherapy cycles than those in the SEMS group, with a median of six (IQR 2–10) vs. two (IQR 2–8) cycles (P < 0.001). There was no significant difference in post-treatment BMI between the two groups (22.5 kg/m^2^ [16.9–25.7 kg/m^2^] vs. 21.6 kg/m^2^ [16.4–26.3 kg/m^2^]; P = 0.375) (**Table 2**). The main reasons for chemotherapy interruption or delay in the SEMS group included persistent or recurrent obstructive symptoms (12 patients), re-obstruction or stent occlusion (10 patients), perforation (2 patients), stent migration (4 patients), poor general condition or nutritional deterioration (8 patients), and disease progression before completion of planned chemotherapy (6 patients). Some patients had more than one reason for treatment interruption.

**Table 2:** Clinical outcomes after treatment models.

Variable	DO (n=62)	SEMS (n=53)	*P* value
CROSS 3 achieved	53(85.5)	25(47.2)	<0.001
Chemotherapy cycles	6 (2-10)	2 (2-8)	<0.001
BMI	22.5 (16.9-25.7)	21.6 (16.4-26.3)	0.375
PNI			0.005
<45	14(22.6)	25(47.2)	
≥45	48(77.4)	28(52.8)	
PLR			<0.001
<162	42(67.7)	19(35.8)	
≥162	20(32.3)	34(64.2)	
NLR			<0.001
<2.5	44(71.0)	20(37.7)	
≥2.5	18(29.0)	33(62.3)	
Response			
Complete response	4(6.5)	2(3.8)	
Partial response	39(62.9)	14(26.4)	
Stable disease	6(9.7)	17(32.1)	
Progressive disease	13(21.0)	19(35.8)	
ORR (%)	43(69.4)	16(30.2)	<0.001
Subsequent resection	43 (69.4)	16 (30.2)	<0.001

PS, performance status; DO, diversion ostomy; SEMS, self-expanding metal stent; CROSS, Colorectal Obstruction Scoring System; PNI, Prognostic Nutritional Index; BMI, Body mass index; PLR, Platelet to lymphocyte ratio; NLR, Neutrophil to lymphocyte ratio.

Regarding nutritional and inflammatory indices after treatment, the DO group exhibited more favorable results. The proportion of patients with PNI ≥ 45 in the DO group was significantly higher than that in the SEMS group (48/62 [77.4%] vs. 28/53 [52.8%]; P = 0.005). Similarly, the proportions of patients with PLR < 162 (42/62 [67.7%] vs. 19/53 [35.8%]; P<0.001) and NLR < 2.5 (44/62 [71.0%] vs. 20/53 [37.7%]; P < 0.001) were both significantly higher in the DO group (**Table 2**).

In terms of tumor response, the DO group had 4 CRs (6.5%), 39 PRs (62.9%), 6 cases of stable disease (9.7%), and 13 cases of progressive disease (21.0%), while the SEMS group had 2 CRs (3.8%), 14 PRs (26.4%), 17 cases of stable disease (32.1%), and 19 cases of progressive disease (35.8%). The objective response rate was significantly higher in the DO group than in the SEMS group [43/62 (69.4%) vs. 16/53 (30.2%), P<0.001]. Consistently, the rate of subsequent resection was significantly higher in the DO group (43/62 [69.4%] vs. 16/53 [30.2%]; P < 0.001) (**Table 2**).

### 3.3 Surgical and pathological findings in patients undergoing subsequent resection

Among patients who subsequently underwent resection, 43 were in the DO group and 16 were in the SEMS group. There was no significant difference in the resection margin status between the two groups (P = 0.804), and the R0 resection rates were 95.3% and 93.8% in the DO and SEMS groups, respectively. Similarly, the pathological response grade did not differ significantly between the groups (P = 0.362) (**Table 3**).

**Table 3:** Surgical and pathological findings after treatments models.

Variable	DO (n=43)	SEMS (n=16)	*P* value
Resection margin			0.804
R0	41(95.3)	15(93.8)	
R1	2(4.7)	1(6.3)	
Pathological response			0.362
0	4(9.3)	3(18.8)	
1	24(55.8)	5(31.3)	
2	8(18.6)	5(31.3)	
3	7(16.3)	3(18.8)	
pT			0.043
T0	4(9.3)	2(12.5)	
T2	1(2.3)	4(25.0)	
T3	8(18.6)	2(12.5)	
T4a	30(69.8)	8(50.0)	
pN			0.987
N0	32(74.4)	12(75.0)	
N1	6(14.0)	2(12.5)	
N2	5(11.6)	2 (12.5)	
N3	0	0	

DO, diversion ostomy; SEMS, self-expanding metal stent.

However, the distribution of pathological T stages differed significantly between the two groups (P = 0.043). In the DO group, the proportions of patients with pT0, pT2, pT3, and pT4a disease were 9.3%, 2.3%, 18.6%, and 69.8%, respectively, while those in the SEMS group were 12.5%, 25.0%, 12.5%, and 50.0%, respectively. In contrast, the distribution of pathological N stages was comparable between the groups (P = 0.987) (**Table 3**).

### 3.4 Survival analysis

**Figure 1:**
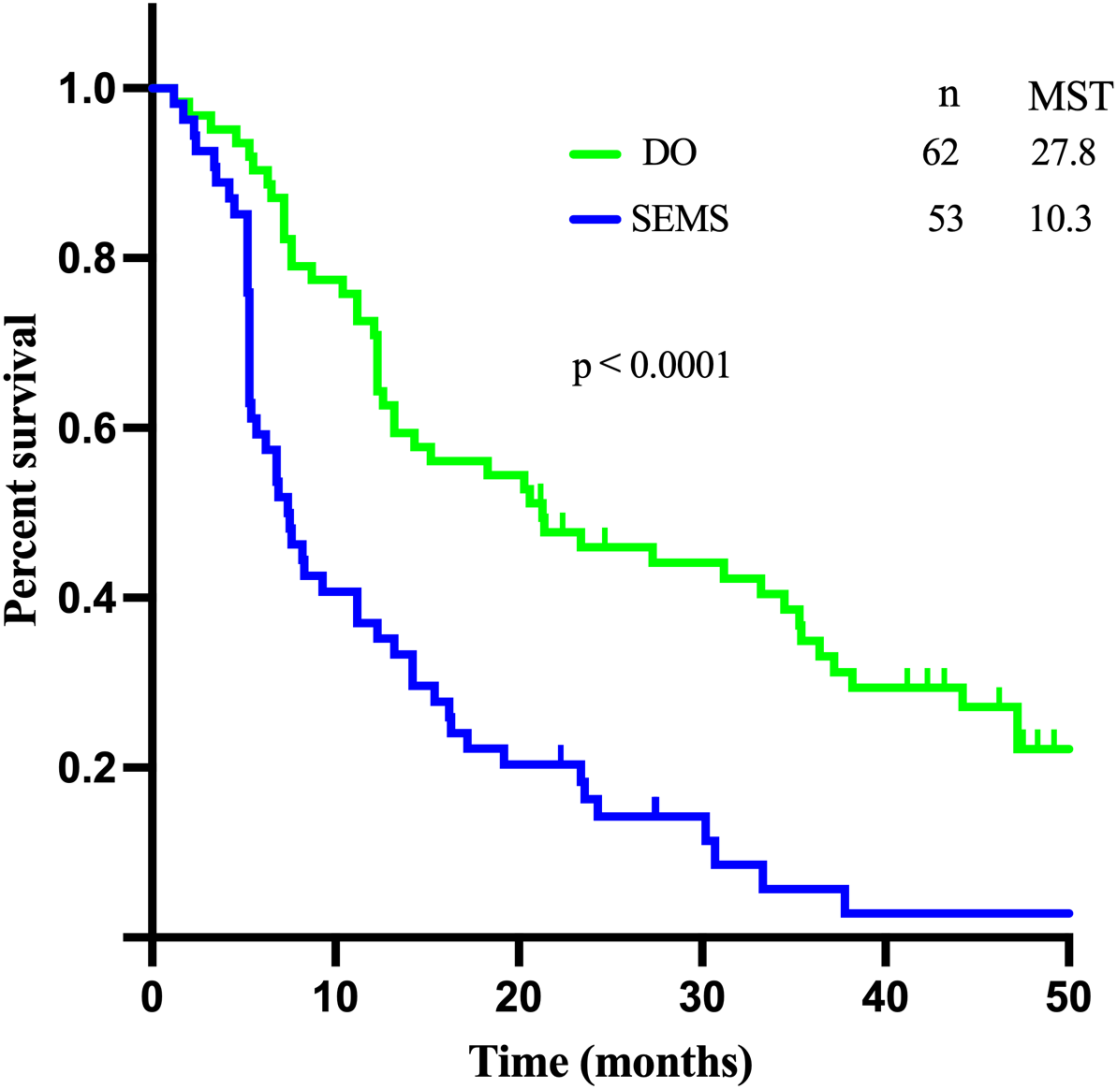
Overall survival according to initial decompression strategy in the overall cohort. DO, diversion ostomy; SEMS, self-expanding metal stent; MST, medium survival time.

**Figure 2:**
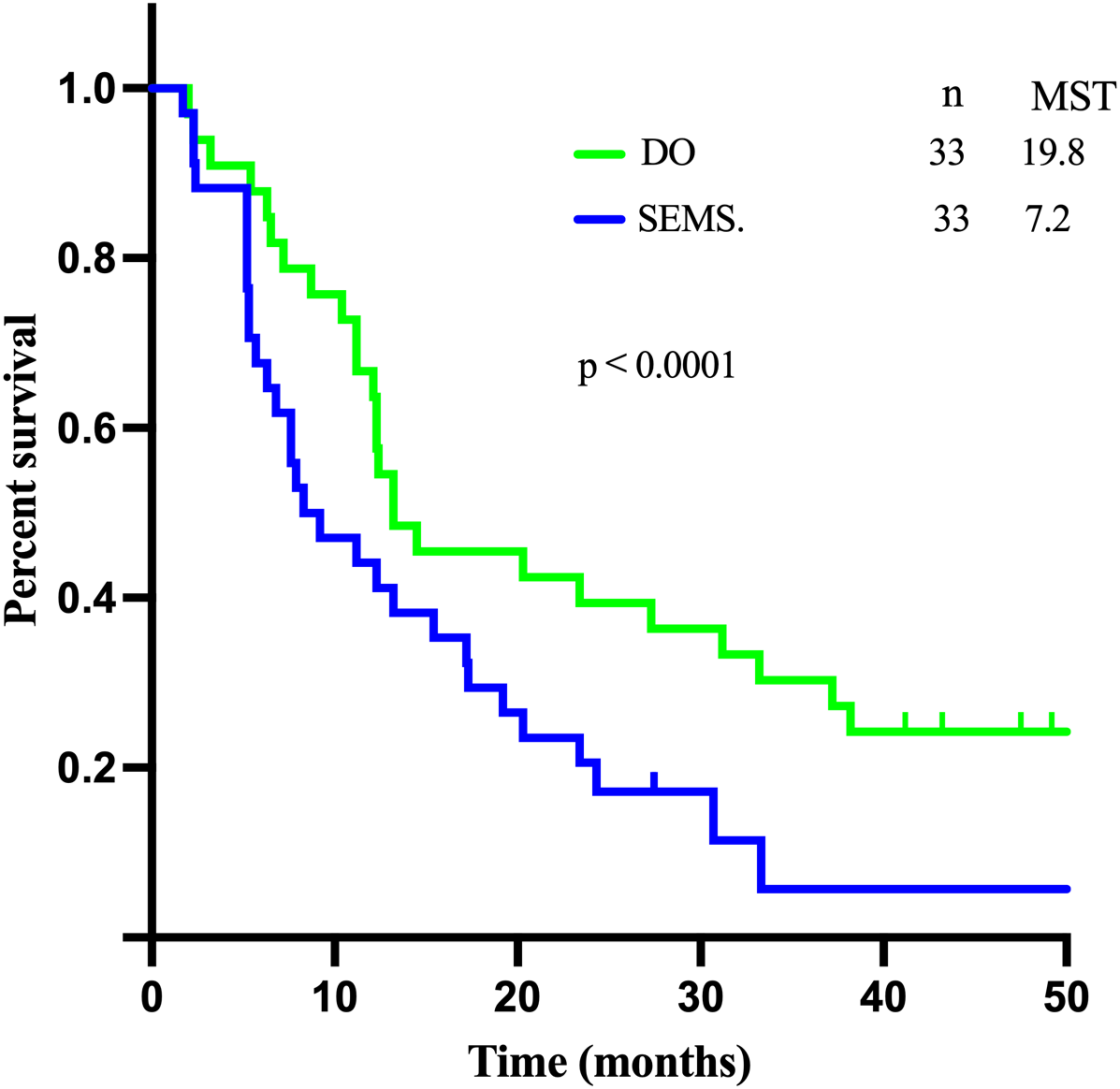
Overall survival according to initial decompression strategy in the propensity score–matched cohort. DO, diversion ostomy; SEMS, self-expanding metal stent; MST, medium survival time.

Kaplan–Meier survival analysis revealed that the DO group experienced significantly better OS than the SEMS group, with a median survival of 27.8 months vs. 10.3 months, respectively (P < 0.0001) (**Figure 1**). In the matched cohort, the survival benefit of DO remained significant. The median survival was 19.8 months in the DO group and 7.2 months in the SEMS group (P<0.0001) (**Figure 2**). Univariate Cox regression analysis identified PS, subsequent resection, and treatment selection as factors associated with OS. Subsequent resection was included in the multivariate analysis as an exploratory prognostic variable because conversion surgery represents a major clinical milestone in this treatment setting. Nevertheless, subsequent resection is likely to be part of the causal pathway from decompression strategy to chemotherapy continuation, conversion to resectability, and improved survival. Therefore, the model including subsequent resection should not be interpreted as proving a direct causal survival benefit of DO independent of the subsequent treatment course. Patients with a PS of 2 experienced worse OS than those with a PS of 0–1 (HR 1.434 [95% CI 1.236–2.176]; P = 0.022). Compared with DO, SEMS treatment was associated with a poorer OS (HR 4.344 [95% CI 1.316–7.569]; P < 0.001). Subsequent resection was also significantly associated with OS (HR 23.431 [95% CI 12.536–66.623]; P < 0.001). Age, sex, BMI, PNI, PLR, and NLR were not significantly associated with OS (**Table 4**).

**Table 4:** Univariate and multivariate analyses for OS.

Variable	Hazard ratio	95% CI	*P* value
Univariate analysis			
Age (≥65/<65)	0.894	0.723-1.221	0.364
Sex (male/female)	1.372	0.987-1.935	0.264
PS (2/0 or 1)	1.434	1.236-2.176	0.022
BMI (≥18.5/<18.5)	1.323	0.734-3.545	0.732
PNI (≥45/<45)	1.123	0.810-1.731	0.737
PLR (≥162/<162)	1.116	0.823-1.213	0.343
NLR (≥2.5/<2.5)	0.732	0.324-1.344	0.876
Subsequent resection (yes/no)	23.431	12.536-66.623	<0.001
Treatment selection(SEMS/DO)	4.344	1.316-7.569	<0.001
Multivariate analysis			
PS (2/0 or 1)	1.643	0.832-2.218	0.341
Subsequent resection (yes/no)	28.943	16.546-81.723	<0.001
Treatment selection(SEMS/DO)	2.231	1.345-3.842	0.001

PS, performance status; DO, diversion ostomy; SEMS, self-expanding metal stent; CROSS, Colorectal Obstruction Scoring System; PNI, Prognostic Nutritional Index; BMI, Body mass index; PLR, Platelet to lymphocyte ratio; NLR, Neutrophil to lymphocyte ratio.

In multivariate analysis, treatment selection and subsequent resection were independently associated with OS. Compared with DO, SEMS placement was associated with a higher risk for mortality (HR 2.231 [95% CI 1.345–3.842]; P = 0.001). Subsequent resection was also significantly associated with OS (HR 28.943 [95% CI 16.546–81.723]; P < 0.001), whereas PS was no longer statistically significant in the multivariate model (HR 1.643 [95% CI 0.832–2.218]; P = 0.341) (**Table 4**).

Subgroup analysis further confirmed the survival advantage of DO over SEMS placement in the overall cohort (P = 0.001), and this association was generally consistent across predefined subgroups, including age, sex, PS, PNI, NLR, and PLR (**Table 5**).

**Table 5:** Subgroup analyses of overall survival according to initial decompression strategy.

Variable		n	HR	95% CI	*P value*
All Patients		n=115	2.23	1.35–3.84	*P<0.0001*
Age	<65	n= 66	2.18	1.27–3.73	*P=0.0040*
	≥65	n= 49	3.05	1.58–5.89	*P=0.0010*
Sex	Male	n= 72	2.76	1.62–4.69	*P<0.0001*
	Female	n= 43	1.68	0.84–3.36	*P=0.1500*
CROSS	0	n= 62	2.15	1.10–4.21	*P=0.0210*
	1	n= 53	3.32	1.70–6.48	*P<0.0001*
PS	0 or 1	n= 45	2.28	1.17–4.44	*P=0.0150*
	2	n= 60	2.06	1.10–3.87	*P=0.0230*
PNI	<45	n= 80	2.89	1.72–4.86	*P<0.0001*
	≥45	n= 35	2.62	1.28–5.37	*P=0.0070*
NLR	<2.5	n= 29	2.71	1.23–5.96	*P=0.0100*
	≥2.5	n= 86	2.64	1.61–4.33	*P<0.0001*
PLR	<162	n= 44	2.31	1.18–4.50	*P=0.0140*
	≥162	n= 71	2.88	1.70–4.89	*P<0.0001*

HR, hazard ratio; CI, confidence interval; DO, diversion ostomy; SEMS, self-expanding metal stent; CROSS, Colorectal Obstruction Scoring System; PS, performance status; PNI, Prognostic Nutritional Index; NLR, neutrophil-to-lymphocyte ratio; PLR, platelet-to-lymphocyte ratio. HRs were calculated for SEMS versus DO. An HR >1 indicates a higher risk of death in the SEMS group and favors the DO-based treatment pathway.

## 4 Discussion

Malignant colonic obstruction in patients with initially non-resectable colorectal cancer is a particularly challenging clinical scenario because the initial local intervention must not only relieve obstruction but also preserve the opportunity for systemic therapy and subsequent conversion surgery. In the present retrospective cohort, the DO-based treatment pathway was associated with a higher rate of restoration to CROSS score 3, more chemotherapy cycles, more favorable post-treatment nutritional and inflammatory indices, a higher objective response rate, a higher rate of subsequent resection, and longer OS than the SEMS-based pathway. However, these findings should not be interpreted as direct evidence of the intrinsic superiority of DO because treatment allocation was strongly influenced by baseline obstruction severity and was not randomized. Rather, our findings describe the outcomes associated with two institutionally selected real-world management pathways.

A key finding of this study was that the advantage of DO was not limited to short-term decompression. Compared with the SEMS group, patients in the DO group were more likely to maintain systemic therapy and undergo resection. This is clinically relevant because in conversion-oriented treatment, the value of decompression lies not simply in bowel relief but also in preserving the entire multidisciplinary treatment sequence. Current guidelines for metastatic colorectal cancer emphasize repeated reassessment of tumor response and resectability during systemic therapy, ideally in an MDT setting, because some initially unresectable tumors may become resectable after adequate downstaging (29–31).The superiority of DO in our cohort may be partially explained by its decompression stability. SEMS have clear advantages, including avoidance of upfront surgery, rapid relief of obstruction, and lower initial invasiveness, and are recommended for selected patients with malignant large bowel obstruction. However, concerns remain regarding long-term patency and complications, such as perforation, migration, and re-obstruction, particularly when prolonged systemic therapy is expected (32, 33). In contrast, diversion surgery is initially more invasive but may provide more durable decompression and reduce the interruption of treatment caused by local luminal failure. In our study, this trade-off appeared to favor DO.

Another important observation was that the DO group exhibited significantly better post-treatment PNI, PLR, and NLR profiles than the SEMS group. This finding is biologically plausible, because persistent or recurrent obstruction may impair oral intake, worsen malnutrition, and perpetuate systemic inflammation. Patients in the DO group received more chemotherapy cycles than those in the SEMS group. This difference may be explained by several factors. First, DO may have provided more stable decompression, thereby improving oral intake, nutritional recovery, and tolerance to systemic therapy. Second, patients in the SEMS group may have been more likely to experience persistent obstructive symptoms, inadequate functional decompression, re-obstruction, or stent-related complications, which could lead to treatment delay or interruption. However, this finding should also be interpreted cautiously because the non-randomized treatment allocation, baseline obstruction severity, patient frailty, physician selection, and survival time bias may have contributed to the observed difference in chemotherapy exposure. Therefore, chemotherapy cycles should be regarded as a treatment-process indicator within the conversion-intent pathway rather than as direct evidence of the intrinsic superiority of DO. Therefore, stable decompression may improve nutritional reserves, reduce the inflammatory burden, and increase the likelihood of completing conversion therapy and reaching surgical reassessment. The higher rate of subsequent resection in the DO group was statistically significant. In modern colorectal cancer management, conversion from non-resectable to resectable remains one of the most important pathways for prolonged survival (34, 35). Because resectability after conversion therapy depends on the treatment response, metastatic burden, technical feasibility, and MDT judgment, our treatment model reflects real-world practice. The finding that DO was associated with both a higher ORR and a higher rate of subsequent resection supports the hypothesis that reliable decompression facilitates the completion of the intended oncological treatment sequence.

Therefore, these findings should be interpreted with caution. First, the two treatment groups were not directly comparable with respect to baseline obstruction severity. All patients in the DO group had a CROSS score of 0, whereas all patients in the SEMS group had a CROSS score of 1. This complete separation reflected our institutional treatment-selection strategy and introduced substantial selection bias. Although baseline PNI, PLR, and NLR distributions did not differ significantly between the groups, comparability in these measured variables does not exclude residual confounding related to age, frailty, disease burden, treatment tolerance, physician preference, or other unmeasured factors. Therefore, the present findings should be interpreted as a comparison of DO-based and SEMS-based treatment pathways rather than as direct causal evidence that one decompression procedure is superior to the other. Second, this was a retrospective single-center study with a limited sample size, and selection and information biases were unavoidable. Third, subsequent resection was a post-treatment variable that likely reflected both treatment response and survival selection. Fourth, the pathological assessment was limited. Important prognostic pathological factors in colorectal cancer, including venous invasion, lymphatic invasion, perineural invasion, and tumor budding, were not consistently available in the original pathological reports and therefore could not be included in the present analysis. The absence of these variables may have limited the ability to fully characterize pathological risk after subsequent resection. Finally, as in many conversion therapy studies, the lack of a universally accepted definition for post-treatment resectability inevitably introduces some subjectivity, although all decisions are made through MDT discussions (10, 11, 13-16).

Despite these limitations, this study focused on a clinically important, but relatively underexplored, subgroup; namely, patients with initially non-resectable colorectal cancer complicated by colonic obstruction and managed with conversion intent. In addition to survival, we evaluated intermediate treatment milestones that may mediate prognosis, including restoration of bowel function, maintenance of chemotherapy, improvement in nutritional and inflammatory status, objective response, and subsequent resection.

## 5 Conclusion

In this retrospective single-center cohort, the DO-based treatment pathway was associated with better decompression, greater chemotherapy exposure, more favorable post-treatment nutritional and inflammatory profiles, a higher subsequent resection rate, and longer OS than the SEMS-based pathway. Because treatment selection was strongly influenced by baseline obstruction severity, these results should not be interpreted as definitive evidence of the superiority of DO over SEMS. Prospective studies with standardized treatment allocation and adequate adjustment for baseline disease severity are required to validate these findings.

## Data Availability

The original contributions presented in the study are included in the article/supplementary material. Further inquiries can be directed to the corresponding author.
